# Ipomoelin, a Jacalin-Related Lectin with a Compact Tetrameric Association and Versatile Carbohydrate Binding Properties Regulated by Its N Terminus

**DOI:** 10.1371/journal.pone.0040618

**Published:** 2012-07-11

**Authors:** Wei-Chieh Chang, Kai-Lun Liu, Fang-Ciao Hsu, Shih-Tong Jeng, Yi-Sheng Cheng

**Affiliations:** 1 Institute of Plant Biology, College of Life Science, National Taiwan University, Taipei, Taiwan, Republic of China; 2 Department of Life Science, College of Life Science, National Taiwan University, Taipei, Taiwan, Republic of China; 3 Technology Commons, College of Life Science, National Taiwan University, Taipei, Taiwan, Republic of China; Bangor University, United Kingdom

## Abstract

Many proteins are induced in the plant defense response to biotic stress or mechanical wounding. One group is lectins. Ipomoelin (IPO) is one of the wound-inducible proteins of sweet potato (*Ipomoea batatas* cv. Tainung 57) and is a Jacalin-related lectin (JRL). In this study, we resolved the crystal structures of IPO in its apo form and in complex with carbohydrates such as methyl α-D-mannopyranoside (Me-Man), methyl α-D-glucopyranoside (Me-Glc), and methyl α-D-galactopyranoside (Me-Gal) in different space groups. The packing diagrams indicated that IPO might represent a compact tetrameric association in the JRL family. The protomer of IPO showed a canonical β-prism fold with 12 strands of β-sheets but with 2 additional short β-strands at the N terminus. A truncated IPO (ΔN10IPO) by removing the 2 short β-strands of the N terminus was used to reveal its role in a tetrameric association. Gel filtration chromatography confirmed IPO as a tetrameric form in solution. Isothermal titration calorimetry determined the binding constants (K_A_) of IPO and ΔN10IPO against various carbohydrates. IPO could bind to Me-Man, Me-Glc, and Me-Gal with similar binding constants. In contrast, ΔN10IPO showed high binding ability to Me-Man and Me-Glc but could not bind to Me-Gal. Our structural and functional analysis of IPO revealed that its compact tetrameric association and carbohydrate binding polyspecificity could be regulated by the 2 additional N-terminal β-strands. The versatile carbohydrate binding properties of IPO might play a role in plant defense.

## Introduction

Plant defense is a complicated mechanism in response to mechanical wounding, herbivore and microorganism attack. Many proteins, namely wound-inducible proteins, are expressed to prevent pathogen infection, inhibit digestion by insects, and repair injured tissues [1], [2]. One group of wound-inducible proteins is lectin, the carbohydrate binding protein [3], [4]. Plant lectins are involved in the plant defense mechanism because of carbohydrate binding properties [5]–[9]. The toxicity of lectins was also confirmed in animal experiments [10], [11]. Plant lectins show resistance to digestive enzymes and can bind selectively to the carbohydrate moieties of gut epithelial cells to interfere in nutrient digestion and absorption [12], so they could be a natural insecticide. In addition, plant lectins have been used for blood typing and immunological assay. The lectin concanavalin A is commercially used in affinity chromatography for purifying glycoproteins. Plant lectins have long been reported as potential inhibitors of viruses [13]–[17].

Most plant lectins were originally isolated from seeds and vegetative storage tissues. Accumulating data have revealed that plants ubiquitously synthesize lectins in response to abiotic and biotic stresses. These inducible lectins are synthesized and then exported to vacuoles by signal peptides or reside in the cytoplasm [18], [19]. The physiological function of plant lectins for subcellular localization remains obscure. However, the major assumption is that lectins are involved in defense and may also have a role in signal transduction for response to stress [20]. Structure analysis of plant lectins demonstrated a diverse group of proteins that can be classified into 6 different groups (http://www.cermav.cnrs.fr/lectines/): monocot lectin, hevein domain lectins, β-prism lectins, β-trefoil lectins, cyanovirin-N homologs, and legume lectin.

**Figure 1:**
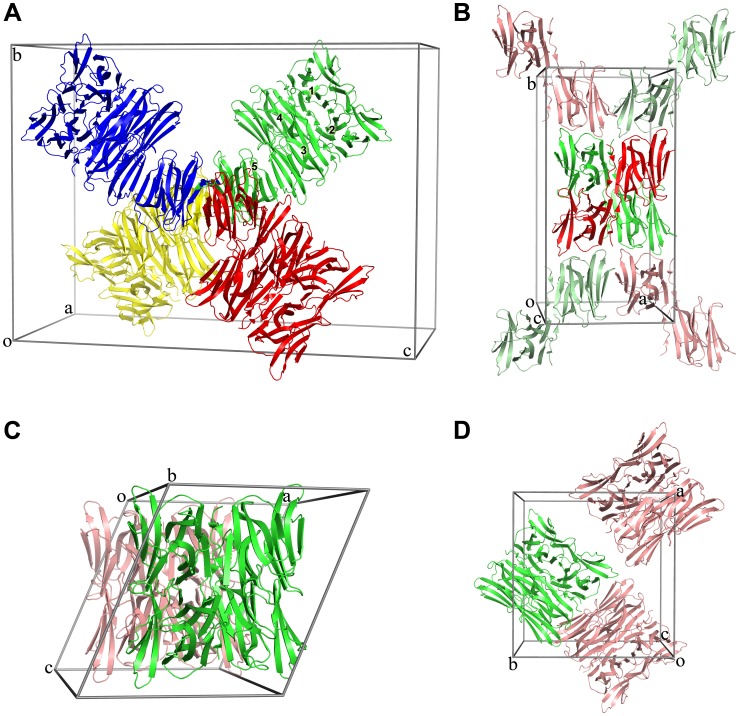
Packing diagram of apo ipomoelin (IPO) (A) and in complex with (B) methyl α-D-mannopyranoside (Me-Man), (C) methyl α-D-glucopyranoside (Me-Glc) and (D) methyl α-D-galactopyranoside (Me-Gal). The resolved IPO structures are in green and the molecules in pink or in light green were generated by symmetric operations. (**A**) The packing diagram of apo IPO with 5 molecules in green. Four of 5 molecules form a tetramer and the 5th molecule can form another tetramer by the red one, the yellow one and the blue one in the center. The molecules in red, yellow and blue are generated by symmetric operations (-X, Y, -Z), (X, -Y, -Z), and (-X-1, -Y, Z). (**B**) The resolved IPO–Me-Man complex in green is 2 molecules in an asymmetric unit. However, the other 2 molecules in red are generated by the symmetric operation (X, -Y, -Z) to form a tetramer in the center. (**C**) The resolved IPO–Me-Glc complex is a tetrameric form, and (**D**) the IPO–Me-Gal complex is also a tetramer. All packing diagrams reveal its tetrameric nature.

Jacalin-related lectins (JRL) have a β-prism fold. In 1996, the structure of Jacalin from seed of jackfruit (*Artocarpus integrifolia*) was first reported to have a tetrameric association for binding to galactose [21]. Later, *Maclura pomifera* seed agglutinin was reported to have the same tetrameric structure as Jacalin [22]. The other lectin, Artocarpin, from seed of jackfruit (*Artocarpus heterophyllus*) shares the same tetrameric association for binding to mannose [23]. Moringa M from black mulberry (*Morus nigra*) forms a tetrameric association like that of Jacalin [24]. JRLs were once thought to be confined to the Moraceae. However, increasing structural evidence reveals that the lectins with a β-prism fold exist universally in plants and animals [25] but with different quaternary association. Heltuba is a plant tuber lectin from *Helianthus tuberosus* (Jerusalem artichoke) that has a donut shape with an octahedral assembly by the β-prism building block [26]. Caselpa is a rhizome lectin from *Calystegia sepium* (Hedge bindweed) that has a dimeric form [27]. PPL is a plant seed lectin from *Parkia platycephala* that contains 3 repetitive β-prism domains and forms a dimeric form with hexahedral assembly [28].

**Figure 2:**
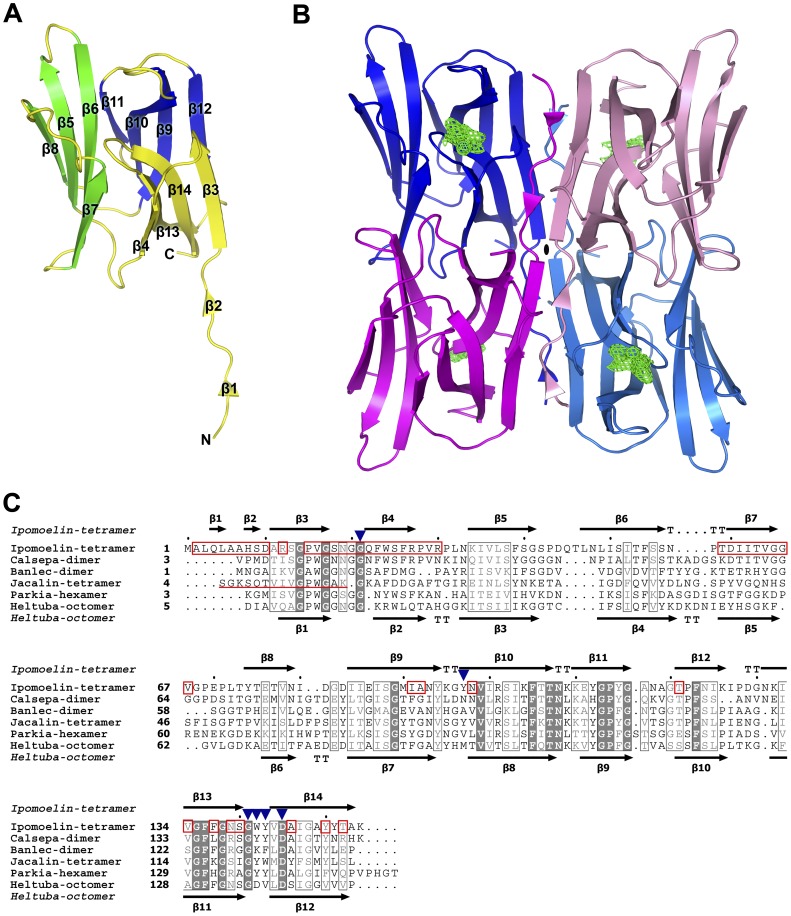
Ribbon diagram of IPO and sequence alignment of Jacalin-related lectins. (**A**) A ribbon diagram of monomeric IPO with 14 β-strands. (**B**) A ribbon diagram of a compact tetrameric IPO. The carbohydrate binding pockets are indicated by green mesh. Tetrameric IPO is represented by monomer A in blue, monomer B in purple, monomer C in light blue, and monomer D in pink. The symmetric axis is represented by a black ellipse in the center of tetramer. (**C**) Structure-based multiple sequence alignment of Jacalin family. Five homologs were selected for sequence comparison from resolved protein structures: Ipomoelin-tetramer from *Ipomoea batatas* (PDB: 3R52); Calsepa-dimer from *Calystegia sepium* (PDB: 1OUW); Banlec-dimer from *Musa acuminate* (PDB: 2BMZ); Jacalin-tetramer from *Artocarpus hirsutus* (PDB: 1TOQ); Parkia-hexamer from *Parkia platycephala* (PDB: 1ZGS); and Heltuba-octomer from *Helianthus tuberosus* (PDB: 1C3K). Positions of identical conserved residues are shown in white on dark grey background, and regions of similarly conserved residues in light grey are boxed. Representation of secondary structure elements and numbering above the alignment is based on the IPO structure. The secondary structure elements below the alignment are based on the Heltuba structure. The carbohydrates Me-Man, Me-Glc, and Me-Gal share 9 hydrogen-bonding interactions with Gly21, Tyr97, Gly141, Trp142, Tyr143 and Asp145 of IPO (blue triangle). The residues of IPO located at the interface are boxed in red. The two short β strands at the N terminus are also involved in the interface. The underlined Jacalin-tetramer representing the sequence is extracted from the C terminus of Jacalin (chain B).

Ipomoelin (IPO), expressed in the leaves of sweet potato (*Ipomoea batatas* cv. Tainung 57), was found easily inducible by wounding and methyl jasmonate [29], [30]. Previous study showed that IPO can agglutinate human blood and bind to different carbohydrates, such as methyl α-D-mannopyranoside (Me-Man), methyl α-D-glucopyranoside (Me-Glc), mannose, glucose and galactose [10]. In this study, we resolved the crystal structures of IPO in the apo form and in complex with Me-Man, Me-Glc and methyl α-D-galactopyranoside (Me-Gal) to reveal the different quaternary associations of IPO and its binding pocket for carbohydrates. A truncated IPO (ΔN10IPO) was prepared to reveal its role in tetrameric association in solution by gel filtration chromatography. In addition, the carbohydrate binding constants of IPO and ΔN10IPO were determined by isothermal titration calorimetry (ITC). ΔN10IPO showed a recovered mannose/glucose-specific lectin. Structural and functional analysis identified IPO as a member of the JRL family but with a different tetrameric association. The N-terminus of IPO plays a critical role in regulating broad carbohydrate binding.

**Figure 3:**
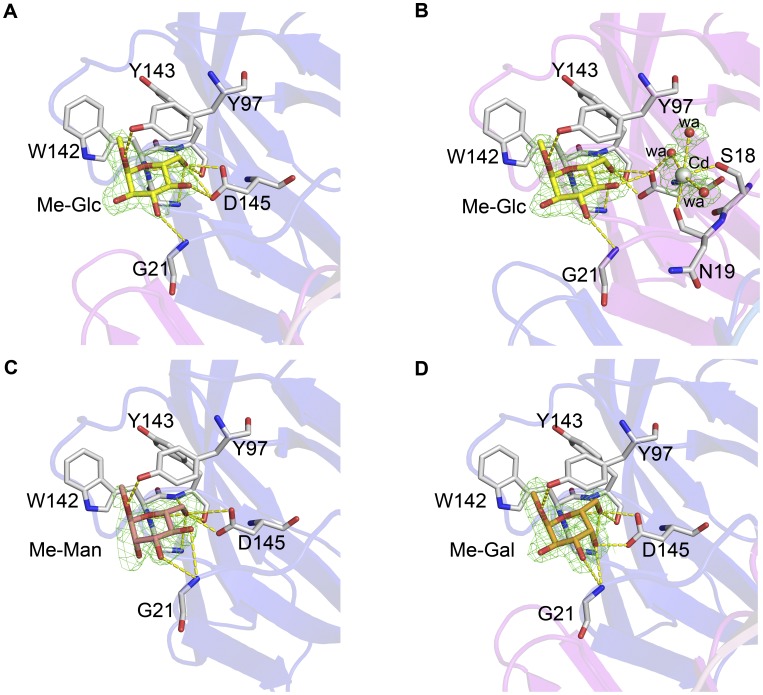
Electron density of carbohydrates from the structures of IPO–Me-Glc and IPO–Me-Man, IPO–Me-Gal. These maps are contoured at 1.0 σ 2fofc electron density. The residues interacting with carbohydrates are highlighted. The carbohydrates Me-Glc only in chain A (**A**), Me-Glc with cadmium ion in chain B–D (**B**); Me-Man (**C**) and Me-Gal (**D**) form the hydrogen-bonding interactions (the yellow dashed lines) with the residues Gly21, Tyr97, Gly141, Trp142, Tyr143 and Asp145 of IPO.

**Table 1:** Interacting residues of IPO with the carbohydrates Me-Glc, Me-Man, and Me-Gal.

IPO	Me-Glc Chain A	Me-Glc Chain B–D	Me-Man	Me-Gal
**Gly21N**	mGlc O3, 3.1 Å	mGlc O3, 3.2 Å	mMan O3, 3.2 Å	mGal O3, 3.0 Å
**Gly21N**	–	–	mMan O4, 3.4 Å	mGal O4, 3.0 Å
**Tyr97OH**	mGlc O1, 3.4 Å	mGlc O1, 3.3 Å	mMan O1, 3.0 Å	mGal O1, 3.2 Å
**Gly141N**	mGlc O6, 3.2 Å	mGlc O6, 3.2 Å	mMan O6, 3.0 Å	mGal O6, 3.4 Å
**Trp142N**	mGlc O5, 2.9 Å	mGlc O5, 3.0 Å	mMan O5, 2.9 Å	mGal O5, 2.8 Å
	mGlc O6, 3.0 Å	mGlc O6, 3.1 Å	mMan O6, 3.0 Å	mGal O6, 3.1 Å
**Tyr143N**	mGlc O6, 2.8 Å	mGlc O6, 2.8 Å	mMan O6, 2.8 Å	mGal O6, 2.7 Å
**Tyr143O**	mGlc O6, 3.1 Å	mGlc O6, 3.1 Å	mMan O6, 3.0 Å	mGal O6, 3.2 Å
**Asp145OD1**	mGlc O6, 2.8 Å	mGlc O6, 3.1 Å	mMan O6, 2.7 Å	mGal O6, 2.8 Å
		mGlc O4, 3.0 Å		
**Asp145OD2**	mGlc O6, 3.0 Å	mGlc O6, 2.7 Å	–	mGal O4, 2.6 Å
	mGlc O4, 3.3 Å	–		

## Results

### Crystal Packings of Apo IPO and IPO–carbohydrate Complexes Show Tetrameric Association

The apo IPO showed an orthorhombic space group of I222. A reasonable volume of the unit cell (Vm) for the Matthew coefficient was estimated at 2.19 Å^3^/Da and 44% solvent content by 8 IPO molecules. However, only 5 IPO molecules in an asymmetric unit could be built after molecular replacement. We obtained a higher Matthew coefficient with 3.51 Å^3^/Da and 65% solvent content. In the packing diagram for apo IPO, we observed a tetrameric association with an additional monomer in an asymmetric unit (Figure 1A). The additional monomer could form a tetrameric association with the other 3 neighboring molecules, which were generated by symmetric operations (-X, Y, -Z), (X, -Y, -Z), and (-X-1, -Y, Z). So 4 IPO molecules could form a tetramer.

**Figure 4:**
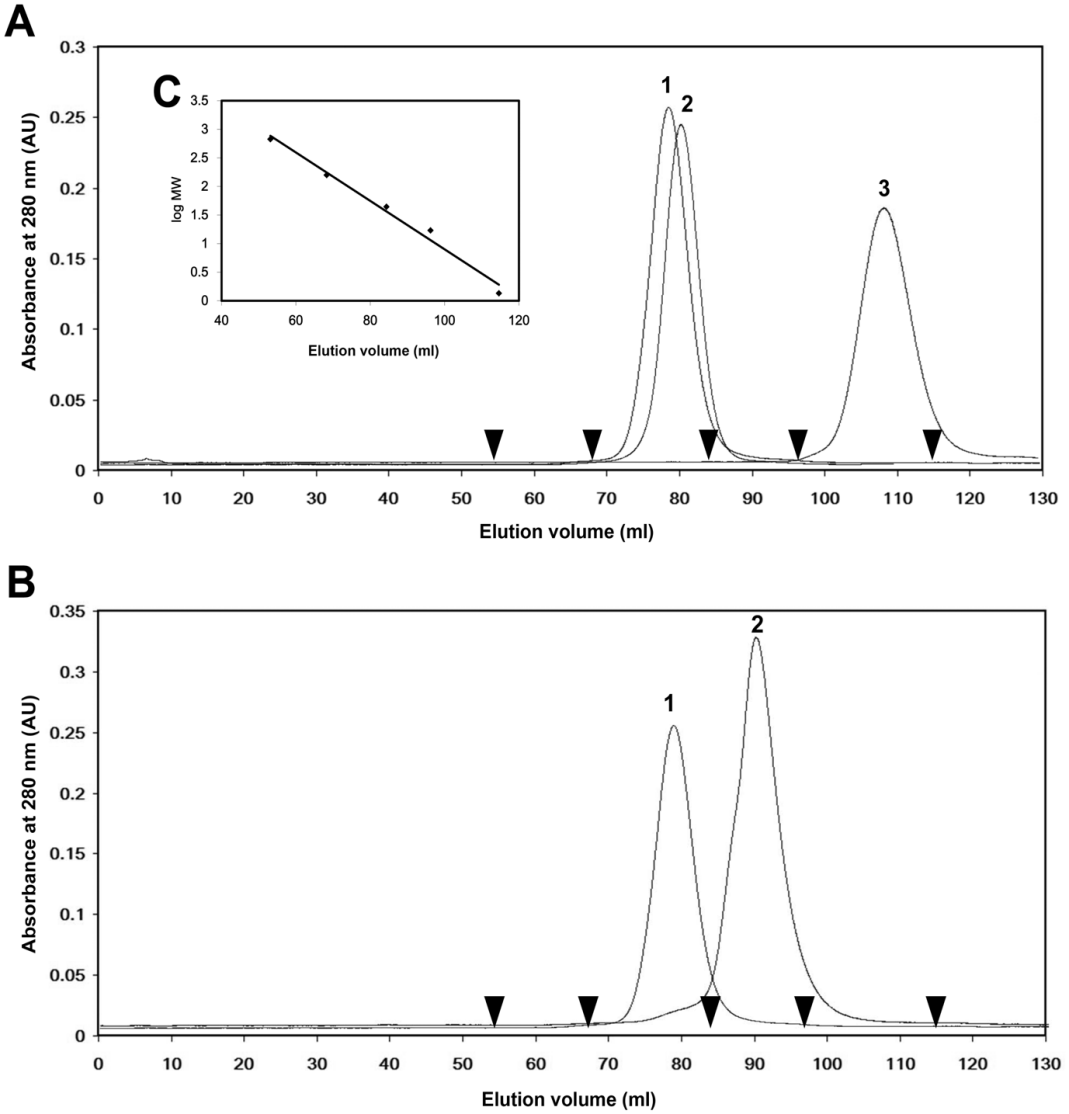
Determination of tetrameric IPO by gel-filtration chromatography. The HiLoad™ 16/60 Superdex™ 200 column was pre-equilibrated with a running buffer containing 27 mM Tris-HCl (pH 7.0) and 2 M NaCl with and without 0.2 M Me-Glc or 1 M glucose at a flow rate of 0.6 ml/min. The elution profiles were monitored at 280 nm. (**A**) Peak 3 represents the IPO protein dissolved in the running buffer without carbohydrates and eluted at 108.2 ml. Peak 2 represents the IPO protein dissolved in the running buffer with 0.2 M Me-Glc and eluted at 80.5 ml. Peak 1 represents the IPO protein dissolved in running buffer with 1 M glucose and eluted at 78.8 ml. The retarded results from peaks 1 and 2 show that the IPO protein could bind to the dextran of the Superdex 200 column. The retarded phenomenon of IPO could be complemented by 1 M glucose. Peak 3 was found with an estimated molecular mass of 63.2 kDa corresponding to a tetramer with 69.2 kDa. (**B**) To determine the role of the N terminus of IPO protein in tetramerization, a truncated IPO by removing residues 1 to 10 was prepared. The proteins were dissolved in the running buffer with 1 M glucose. Peak 1 represents the native IPO and was eluted at 78.8 ml. Peak 2 represents the truncated IPO and was eluted at 89.6 ml. Peak 2 was calculated with a molecular mass of 22.0 kDa corresponding to a truncated monomer with 16.3 kDa. (**C**) The standard markers from BioRad containing thyroglobulin (670 kDa), gamma-globulin (158 kDa), ovalbumin (44 kDa), myoglobin (17 kDa), and vitamin B12 (1.35 kDa) were used to calculate the equation of linear regression. The X-axis represents the elution volume and Y-axis the log value of molecular mass from the standard markers. The equation is y = -0.0423x+5.1333 and R^2^ = 0.9847.

To determine the carbohydrate binding pocket of IPO, carbohydrates such as Me-Man, Me-Glc and Me-Gal were used to co-crystallize with the IPO protein. The crystals of IPO–carbohydrate complexes were determined in different space groups. IPO–Me-Man belongs to an orthorhombic space group C222_1_. The Matthew coefficient and solvent content for IPO–Me-Man had a reasonable value of 2.21 Å^3^/Da and 44.4% for 2 molecules in an asymmetric unit. Although only 2 IPO molecules were built in the IPO–Me-Man complex, the other 2 IPO molecules could be generated by symmetric operation (X, -Y, -Z) and resulted in a tetrameric association (Figure 1B). The crystal of IPO–Me-Glc was determined to be a monoclinic space group P2_1_. The Matthews coefficient and solvent content was 2.26 Å^3^/Da and 45.5% for 4 molecules. The packing results for IPO–Me-Man and IPO–Me-Glc indicated that the carbohydrates binding to IPO might result in a compact packing as compared with that of apo IPO. In addition, the resolved structure of IPO–Me-Glc formed a tetrameric association (Figure 1C).

**Table 2:** Thermodynamics values of IPO and ΔN10IPO titrated with various carbohydrates*.

ProteinCarbohydrates	*c* value	n^a^	*K*_A_(10^3^ M^−1^)	ΔH(kcal/mol)	ΔG(kcal/mol)	-TΔS(kcal/mol)
**IPO**						
Me-Man	7.04	1.0	7.04(±0.20)	−5.56(±0.28)	−5.17(±0.15)	0.39(±0.31)
Me-Glc	2.01	1.0	2.01(±0.05)	−4.12(±0.11)	−4.57(±0.12)	−0.45(±0.02)
Me-Gal	4.09	1.0	4.09(±0.06)	−5.14(±0.13)	−4.94(±0.02)	0.20(±0.12)
Man	0.32	1.0	0.11(±0.00)	−4.61(±0.14)	−2.75(±0.08)	1.86(±0.06)
Glc	0.10	1.0	0.03(±0.00)	−3.17(±0.10)	−2.04(±0.06)	1.13(±0.03)
Gal	0.17	1.0	0.06(±0.00)	−4.78(±0.15)	−2.39(±0.07)	2.39(±0.07)
**ΔN10IPO**						
Me-Man	18.97	1.0	37.94(±1.18)	−13.64(±0.33)	−5.99(±0.72)	7.66(±0.75)
Me-Glc	6.80	1.0	13.60(±0.12)	−9.53(±0.25)	−5.57(±0.01)	3.97(±0.10)
Me-Gal	ND	–	–	–	–	–

* Triple repeats were analyzed and the values represented the average with standard errors in parenthesis.

a The n value was fixed at 1.0 for fitting the curves.

ND represents not determined.

**Figure 5:**
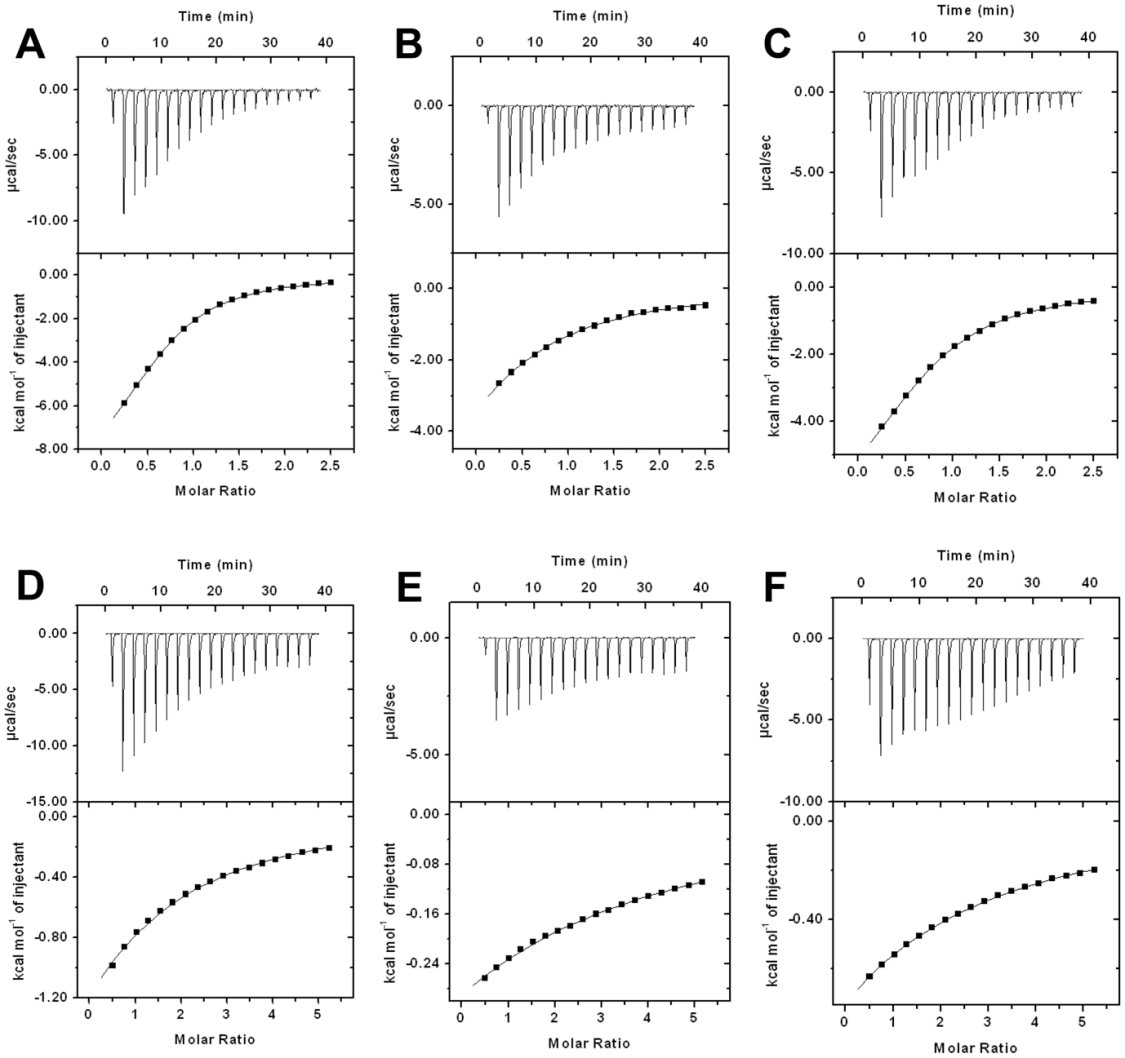
ITC binding assay of wildtype IPO (tetremeric IPO) with carbohydrates. For the methylated carbohydrates, the thermal changes were detected with 1 mM wildtype IPO which was titrated by 25 mM Me-Man (**A**), 25 mM Me-Glc (**B**), 25 mM Me-Gal (**C**). For the non-methylated carbohydrates, the thermal changes were detected with 3 mM IPO which was titrated by 75 mM Man (**D**), 75 mM Glc (**E**) and 75 mM Gal (**F**). The upper panel of figures was presented by 18 injections and 2 µl/per injection. The interval of injection time is 180 sec. The 18 experimental data were almost fitted for a 1∶1 binding model (one-site of fitting) with Microcal Origin 7.0 software (the bottom panel). In each bottom panel, X-axis indicates the molar ratio of protein-carbohydrate and Y-axis indicates the thermal change in each injection.

IPO–Me-Gal belongs to an orthorhombic space group P2_1_2_1_2_1_. The Matthews coefficient and solvent content were 2.25 Å^3^/Da and 45.2%, respectively, for 4 molecules in an asymmetric unit. The 4 IPO–Me-Gal molecules shown in Figure 1D form the same tetrameric association as that of IPO–Me-Glc. On the basis of crystal packings of apo IPO and IPO–carbohydrate complexes, IPO would form a tetrameric association.

**Figure 6:**
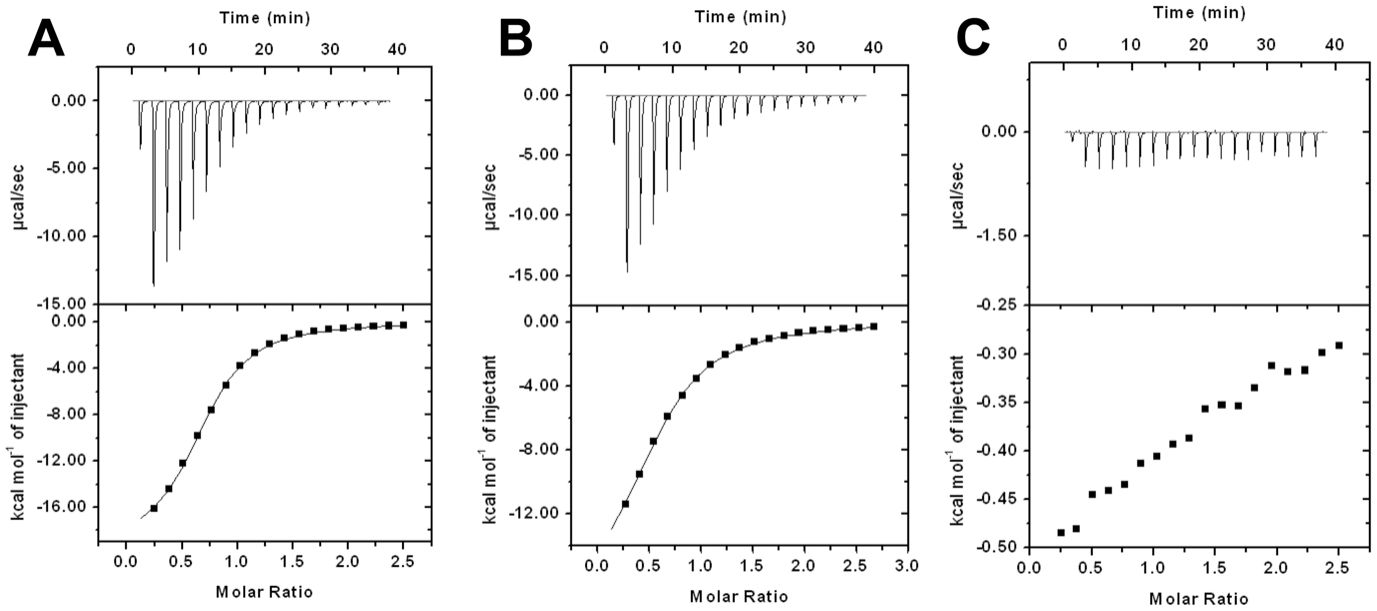
ITC binding assay ofΔN10IPO (monomeric IPO) with methylated carbohydrates. The thermal changes were detected by 0.5 mM ΔN10IPO with 12.5 mM Me-Man (**A**), 0.75 mM ΔN10IPO with 20 mM Me-Glc (**B**) and 1 mM ΔN10IPO with 25 mM Me-Gal (**C**). The upper panel of figures was presented by 18 injections and 2 µl/per injection. The interval of injection time is 180 sec. The 18 experimental data were almost fitted for a 1∶1 binding model (one-site of fitting) with Microcal Origin 7.0 software (the bottom panel). In each bottom panel, X-axis indicates the molar ratio of protein-carbohydrate and Y-axis indicates the thermal change in each injection. In the titration of ΔN10IPO with Me-Gal, no obvious thermal changes could be detected.

### Overall Structure of Monomeric IPO and its Tetrameric Association

The monomeric IPO from residues 1 to 154 shows a typical β-prism fold found in the JRL family, with 12 β-sheets (β3-β14) and 2 additional short, extended, N-terminal β-strands (β1-β2) (Figure 2A and 2C). Each β-prism fold comprises 3 Greek-key motifs forming 3 planes by 3 four-stranded β-sheets: plane 1 by β3 to β4 and β13 to β14; plane 2 by β5 to β8; plane 3 by β9 to β12. Furthermore, the structure of these β-sheets comprises β1 from residues Gln4 to Leu5, β2 from residues His8 to Ser9, β3 from residues Ala11 to Gly17, β4 from residues Gln22 to Arg27, β5 from residues Lys34 to Gly41, β6 from residues Leu47 to Ser55, β7 from residues Ile61 to Gly65, β8 from residues Tyr74 to Asn79, β9 from residues Ile84 to Tyr94, β10 from residues Tyr97 to Thr107, β11 from residues Glu111 to Gly116, β12 from residues Thr121 to Lys126, β13 from residues Asn131 to Ser140, and β14 from residues Val144 to Ala153 (Figure 2A and 2C).

**Figure 7:**
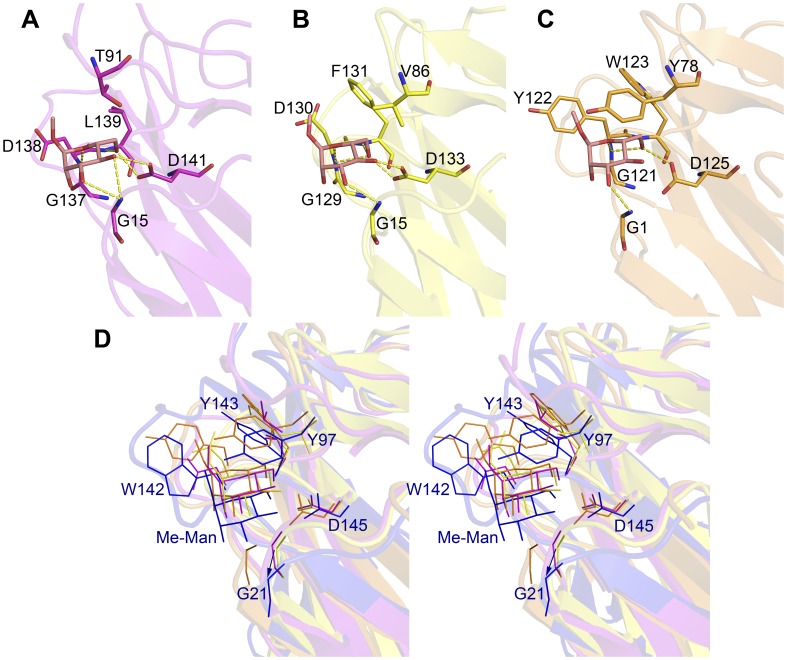
Comparison of interacting residues with Me-Man in binding pocket of Artocarpin, Banlec, Jacalin and IPO. (**A**) The interacting residues of Artocarpin (PDB: 1J4U) with Me-Man are Gly15, Thr91, Gly137, Asp138, Leu139, and Asp141. Sticks of Artocarpin are purple. (**B**) The interacting residues of Banlec (PDB: 1X1V) with Me-Man are Gly15, Val86, Gly129, Asp130, Phe131, and Asp133. Sticks of Banlec are yellow. (**C**) The interacting residues of Jacalin (PDB: 1WS5 chain A) with Me-Man are Gly1, Tyr78, Gly121, Tyr122, Trp123, Asp125. Sticks of Jacalin are orange. (**D**) Stereo view of the binding pocket of Artocarpin, Banlec, Jacalin, and IPO with Me-Man. The structures of Artocarpin (in purple), Banlec (in yellow), Jacalin (in orange) and IPO (in blue) were superimposed. The binding pocket of IPO is labeled with the interacting residues Gly21, Tyr97, Gly141, Trp142, Tyr143 and Asp145, with Me-Man in blue. The binding mode of Me-Man in IPO is compared with the other 3 lectins and are shifted outside by hydrogen binding with Gly21. The moving distance is about 1.5 Å (indicated by an arrow) from Gly15 of Artocarpin and Banlec to Gly21 of IPO.

Four IPO protomers form a compact tetrameric association by swapping their extended N termini from residues 1 to 10. We analyzed the tetrameric association of IPO–Me-Glc. As shown in Figure 2B, the 2 extended N termini from monomer A in blue and monomer B in purple swap with each other. The interacting interface between the four IPO protomers is formed by the extended N termini. Consequently, a larger buried interface between monomers A and B is 1,522 Å^2^. The residues located at the interface are 2–10, 12, 15–30, 59–67, 91–92, 98, 121, 134, 137, 139–140, 146, 150, and 152 in monomer A (as shown in red box in Figure 2C). In total, 13 hydrogen bonds are formed by the residues Leu5, His8, Asn19, Gln22, Ser25, Arg27, Asp60, Ile61, Thr63, Thr121, Asn139 and Tyr150 in the interface between monomers A and B. The buried interface between monomer C and monomer D is 1,554 Å^2^. Furthermore, the buried interface between monomers A and C is 755 Å^2^, which is mainly contributed by the interacting residues of N-terminal residues 4 to 17 and C-terminal residues 91, 121–126, 128, and 151. In addition, the interface between monomers D and B is 731 Å^2^.

### The Carbohydrate Binding Pocket of IPO

The carbohydrate binding pocket of IPO was confirmed at loops β13 and β14 by the structures of IPO–Me-Glc, IPO–Me-Man and IPO–Me-Gal (as shown in Figure 2B with green mesh). In the chain A of IPO–Me-Glc, 9 hydrogen bonds are formed by the residues Gly21, Tyr97, Gly141, Trp142, Tyr143 and Asp145 of IPO and the atoms O1, O3, O4, O5, and O6 of Me-Glc (Figure 3A and Table 1). The atom C7 of Me-Glc is involved in the methyl carbon (Me)…π interaction with Trp142 of IPO. The hydrogen bonds are slightly different between chain A and chains B to D. The hydrogen bonds of chains B to D are formed between the same residues of chain A and Me-Man, except for one additional bonding from Asp145 of IPO and O4 of Me-Glc (Table 1). The differences might result from the binding of cadmium ion (Cd^2+^). In chains B to D, the Cd^2+^ atom forms 5 coordinates by the O atom of the carbonyl group of Asn19, OG atom of Ser18, and 3 water molecules. One of the 3 water molecules forms a hydrogen bond with Asp145 (Figure 3B).

**Figure 8:**
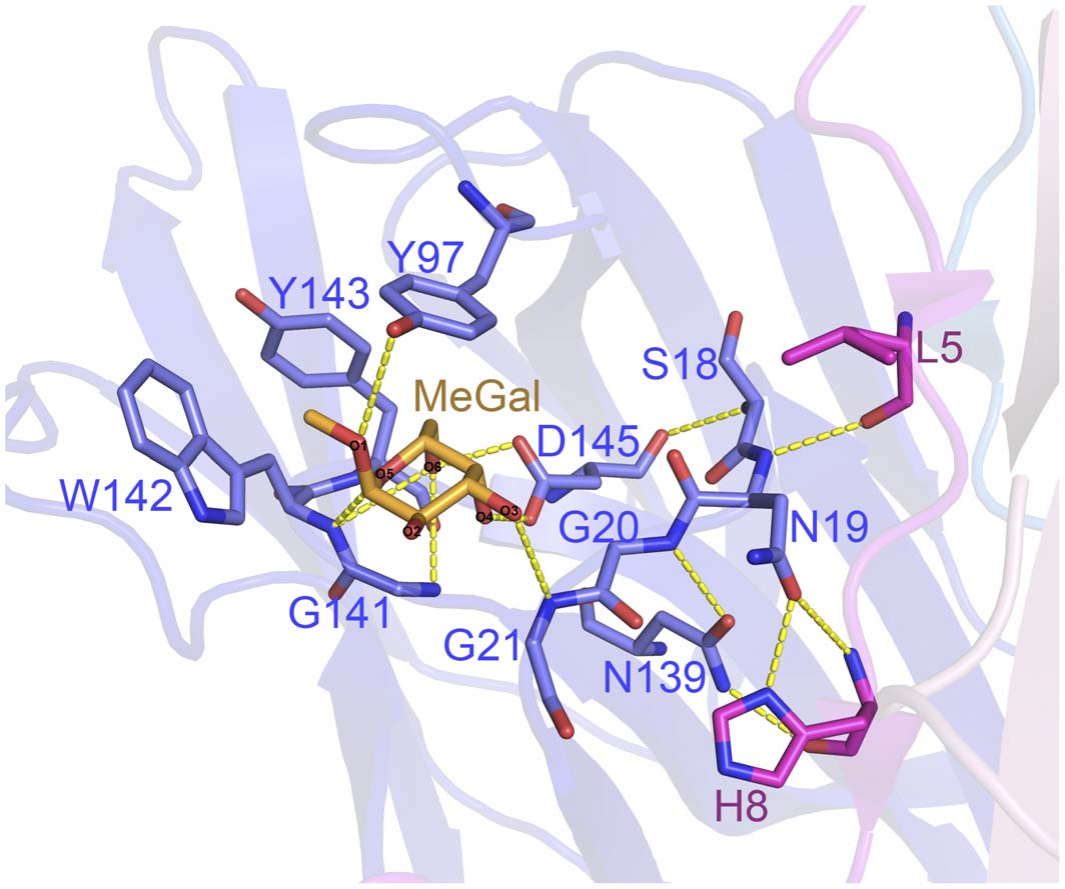
Hydrogen bond networks between carbohydrate binding pocket of chain A and N terminus of chain B. The carbohydrate binding pocket of chain A is shown in blue, and the N terminus of chain B is shown in magenta. The monosaccharide Me-Gal is orange. Four hydrogen bonds are formed: 2 by the atom OD1 of Asn19 (chain A) and the atoms ND1 and N of His 8 (chain B); 1 by the atom N of Asn19 (chain A) and the atom O of Leu5 (chain B); and 1 by the atom ND2 of Asn139 (chain A) and the atom O of His8 (chain B).

In the structure of IPO–Me-Man, two IPO protomers were built, and only one Me-Man molecule could be observed in chain A. The temperature factor of Me-Man in the structure of IPO–Me-Man is 66.5 Å^2^, which is higher than that of Me-Glc, with 34.5 Å^2^ (Table 1). This phenomenon might indicate that only a few Me-Man molecules bound to IPO proteins in IPO–Me-Man, which resulted in a higher temperature factor. Nine hydrogen bonds are formed by the residues Gly21, Tyr97, Gly141, Trp142, Tyr143, and Asp145 of IPO and the atoms O1, O3, O4, O5 and O6 of Me-Man (Figure 3C and Table 1). The atom C7 of Me-Man is also involved in the Me…π interaction with Trp142 of IPO. The binding orientation of Me-Man is similar to that of Me-Glc. In the structure IPO–Me-Gal, 10 hydrogen bonds are formed by the same residues Gly21, Tyr97, Gly141, Trp142, Tyr143, and Asp145 of IPO (Figure 3D and Table 1). The atom C7 of Me-Gal is shown in the Me…π interaction with Trp142 of IPO. This revealed the importance of the methyl group of carbohydrates for binding to IPO.

### The Tetrameric Form of IPO Identified by Gel Filtration Chromatography

To validate that the quaternary association of IPO is also a tetrameric form in solution, purified IPO was used in gel filtration experiments. The molecular mass of IPO could be calculated according to the linear regression equation of the standard protein markers purchased from BioRad (Figure 4C). In the preliminary study, IPO protein was dissolved in running buffer (27 mM Tris-HCl pH 7.0, 2 M NaCl) without additional carbohydrates. We obtained a retarded result, with corresponding molecular mass 4.0 kDa (Peak 3 in Figure 4A). Thus, IPO has the binding ability of dextran in the matrix of the Superdex 200 column. To eliminate the binding effect of IPO to dextran, running buffer was prepared with an additional 0.2 M Me-Glc, and a shift of the IPO peak could be observed, with corresponding molecular mass of 53.3 kDa (Peak 2 in Figure 4A). Consequently, running buffer with an additional 1 M glucose was prepared to totally eliminate the binding effect of IPO. The corresponding molecular mass of IPO in solution was 64.7 kDa (Peak 1 in Figure 4A). The molecular mass of recombinant IPO with a His tag was 17.3 kDa for a monomer and 69.2 kDa for a tetramer. The results from gel filtration experiments demonstrated that IPO shows a tetrameric association in solution.

To further identify the role of the N terminus in the tetramerization of IPO, we prepared a truncated IPO (ΔN10IPO) by removing residues 1 to 10 to monitor the change in quaternary association. The native IPO protein or the truncated IPO protein was dissolved in the running buffer with 1 M glucose. Peak 1 in Figure 4B represents the native IPO, with molecular mass 63.2 kDa, which is a tetrameric size. Peak 2 in Figure 4B represents the ΔN10IPO, with molecular mass 21.9 kDa, which is near the truncated monomer size (16.3 kDa). The results further confirmed that IPO has a tetrameric association and its N terminus plays an important role in forming a tetramer.

### Binding Constants of IPO and Truncated IPO to Various Carbohydrates Detected by ITC

To determine the binding constants of IPO to Me-Man, Me-Glc and Me-Gal, 1 mM IPO solution was titrated with 25 mM carbohydrate solution. The interaction of IPO and carbohydrate was an exothermal reaction. The optimal curves and thermodynamics parameters could be fitting and calculated by Microcal Origin 7.0. The K_A_ of IPO to Me-Man was the highest, 7.04×10^3^ M^−1^. The K_A_ values for Me-Gal and Me-Glc were 4.09×10^3^ M^−1^ and 2.01×10^3^ M^−1^, respectively (Table 2 and Figure 5).

Subsequently, carbohydrates without the methyl group were used to determine the binding affinity of IPO. From preliminary study, 1 mM IPO titrated with 25 mM Man, Glc, and Gal revealed no obvious exothermal reaction. After increasing the concentration with 3 mM IPO titrated with 75 mM Man, Glc, and Gal, the exothermal curves could be observed and calculated. The K_A_ values for IPO binding to Man, Gal and Glc were 1.05×10^2^ M^−1^, 0.57×10^2^ M^−1^, and 0.32×10^2^ M^−1^ (Table 2 and Figure 5). Thus, the interactions between IPO and carbohydrates were stronger with than without the methyl group.

**Table 3:** Crystallography statistics for apo ipomoelin (IPO) and IPO in complex with carbohydrates methyl α-D-mannopyranoside (Me-Man), methyl α-D-glucopyranoside (Me-Glc) and methyl α-D-galactopyranoside (Me-Gal).

Crystals	apo IPO	IPO–MeMan	IPO–MeGlc	IPO–MeGal
Beamline	BL13B1	BL13B1	BL13C1	BL13C1
Wavelength (Å)	1.000	1.000	0.97622	0.97622
***Data collection and processing***				
Space group	I222	C222_1_	P2_1_	P2_1_2_1_2_1_
Cell dimensions (Å and deg.)	87.5, 139.5, 189.9; 90°,90°, 90°	59.9, 118.1, 82.6; 90°, 90°,90°	59.3, 83.7, 65.1; 90°, 112.8°,90°	82.1,83.9,86.1; 90°,90°,90°
Resolution (Å; last shell)	30.0−2.27 (2.35−2.27)	30.0−2.10 (2.18−2.10)	30.0−2.10 (2.18−2.10)	30.0−1.9 (1.97−1.9)
Completeness (%; last shell)	96.6 (99.9)	99.5 (100.0)	97.0 (93.5)	100.0 (100.0)
<*I/σ*(*I*)> (last shell)	18.8 (4.6)	21.0 (6.1)	16.3 (3.6)	29.9 (7.6)
Total reflections	379,028	199,784	104,005	347,548
Unique reflections	51,599	17,358	33,243	47,849
*R_merge_ (%; last shell)	8.2 (45.7)	12.6 (49.5)	6.4 (26.9)	6.7 (30.3)
***Refinement***				
Resolution range (Å)	30.0−2.27	30.0−2.10	30.0−2.10	30.0−1.9
Reflections (working/test)	46,745/2,472	15,052/1,662	28,952/3,216	45,286/2,288
Protein atoms	5,794	2,307	4,625	4,667
Solvent atoms	370	217	473	292
R-factor (%)	20.2	18.6	18.8	17.8
R_free_ (%)	23.4	24.6	24.4	22.7
***Model quality***				
RMS deviations in				
Bond length (Å)	0.015	0.012	0.010	0.024
Bond angle (deg.)	1.7	1.7	1.5	2.1
Average B-factor (Å^2^)				
B-factor (protein)	35.3	28.1	26.0	18.8
B-factor (water)	37.6	39.3	35.0	22.0
B-factor (sugar)		66.5	34.5	15.7
Ramachandran plot (%)				
Most favored	86.9	85.5	87.2	93.5
Additionally allowed	12.3	13.6	10.9	5.4
Generously allowed	0.8	0.4	1.4	1.0
Disallowed	0.0	0.4	0.4	0

* *R*_merge_ = Σ*_h_*Σ*_i_*|*I_h,i_*-<*I_h_*>|/Σ_h_Σ_i_ *I_h,i_* where <*I_h_*> is the mean intensity of *i* observations for a given reflection *h*.

ΔN10IPO was used to determine the role of the N terminus of IPO in binding to carbohydrates. ΔN10IPO at 0.5, 0.75 and 1 mM was titrated with 12.5 mM Me-Man, 20 mM Me-Glc, and 25 mM Me-Gal, respectively. Interestingly, no exothermal was observed with titration of ΔN10IPO to Me-Gal. The K_A_ value for ΔN10IPO binding to Me-Man and Me-Glc was 3.79×10^4^ M^−1^ and 1.36×10^4^ M^−1^, respectively (Table 2 and Figure 6). Thus, the N-terminus of IPO is involved in tetramerization in regulating the binding affinity to carbohydrates.

## Discussion

### Various Quaternary Structures in the JRL Family

We submitted the coordinates of a monomer of apo IPO (e.g., chain A; Figure S1C) to the web service Matras for 3-D protein structure comparison [31]. We found the highest Z-score, 124.5, for the template structure, a dimeric form of Calsepa from *Calydyrgia sepium* (PDB: 1OUW; Figure S1D) [27], in our molecular replacement procedure. The following structures were PPL from *Parkia platycephala* with a hexahedral ring (PDB: 1ZGR; Figure S1E) [28], Heltuba from *Helianthus tuberosus* with an octahedral ring (PDB: 1C3K; Figure S1F) [26], Banlec from banana with an another kind of dimeric form (PDB: 2BMZ; Figure S1B) [32], and Jacalin from jackfruit seeds with a tetrameric form (PDB:1UGW; Figure S1A) [33]. These data indicate the various quaternary structures in the JRL family, despite the same β-prism fold of protomer.

The various quaternary associations in the JRL family exhibited different contacts between protomers. A previous report indicated that the buried interface of the Calsepa dimer is 1,327 Å^2^ by a probe with 1.6 Å radius [27]. Here, we analyzed the buried interface of the selected structures from the above comparison by using the PDBe PISA service with 1.4 Å radius [34]. The buried interface area from tetrameric IPO encompasses 1,539 Å^2^, which is larger than that of Calsepa (1,202 Å^2^), PPL (1,294 Å^2^), Banlec (750 Å^2^), Heltuba (736 Å^2^), and Jacalin (1023 Å^2^). The N terminus of the protomer in the JRL family has an important role in the quaternary association by swapping in the interface and then forming a dimer, tetramer, hexamer, and octomer. To compare the difference between the tetrameric Jacalin (Figure S1A) and the tetrameric IPO (Figure S1C), the tetramer of Jacalin showed a looser interface than that of IPO. Therefore, IPO formed a different compact tetramer.

### The Carbohydrate Binding Pocket of IPO Reveals its Versatile Binding Properties

In this study, we resolved the crystal structures of IPO–Me-Man, IPO–Me-Glc and IPO–Me-Gal complexes. These monosaccharides showed similar orientation to bind to IPO. The binding pocket of IPO contains 6 residues such as Gly21, Tyr97, Gly141, Trp142, Tyr143 and Asp145, to form hydrogen bonds with different monosaccharides (Figure 3). Me-Man and Me-Glc are epimers differing only at the C2 position, and IPO has no hydrogen bonds for C2 atom. Me-Man and Me-Glc share similar binding properties for IPO. However, Me-Gal and Me-Glc are epimers at the C4 position. The C4 atom of Me-Glc and Me-Gal could form one hydrogen bond with the amine group of Gly21 of IPO and one hydrogen bond with the β-carboxylic group of Asp145 of IPO (Figure 3A and Figure 3D). From the affinity binding results from ITC, K_A_ values for IPO to Me-Man, Me-Gal and Me-Glc range from 7.04×10^3^ M^−1^ to 2.01×10^3^ M^−1^ (Table 2). The carbohydrate binding manner of IPO is not confined as is the mannose- glucose-specific binding lectin.

In addition to determining monosaccharides with the methyl group, we used monosaccharides without a methyl group, such as mannose (Man), glucose (Glc), and galactose (Gal), to determine their binding constant to IPO. Since the lower binding affinity of IPO titrated with Man, Glc or Gal couldn’t get the best fitting for the titration curves, the n value was consequently fixed at 1.0 for fitting the curves (Table 2). K_A_ values for IPO to Man, Glc and Gal ranged from 0.3×10^2 ^M^−1^ to 1.1×10^2 ^M^−1^, for about one-thirtieth those of monosaccharides with methyl group. The difference is just from a methyl group. After examining the IPO–methyl monosaccharide complex structures, the methyl group of monosaccharide oriented toward the indole group of Trp142 and formed the nonpolar interaction of Me…π. Therefore, the methyl group of monosaccharides would have an important interaction force to bind to IPO.

Up to now, the binding constants of the lectins such as Artocarpin, Banlec, CCA, PAL, and Jacalin, of the JRL family have been determined by ITC. K_A_ values for Artocarpin to Me-Man and Man are 2.5×10^3^ M^−1^, 1.64×10^3^ M^−1^, and to Me-Glc and Glc are 3.41×10^2^ M^−1^, 1.5×10^2 ^M^−1^, respectively [35]. K_A_ values for Banlec to Me-Glc and Glc are 1.3×10^2^ M^−1^, 1.22×10^2^ M^−1^ [36]. The results show no differences with or without the methyl group of monosaccharides for binding properties in Artocarpin and Banlec possibly because of no aromatic side chain of residues in Artocarpin and Banlec like the residue Trp142 in IPO (Figure 7A and 7B). Interestingly, IPO shared similar binding properties to Jacalin for its Tyr122, which might interact with the methyl group of monosaccharides (Figure 7C). K_A_ values of Jacalin to Me-Gal and Gal are 21.22×10^3^ M^−1^ and 0.8×10^3^ M^−1^ and to Me-Man and Man 1.08×10^3^ M^−1^ and 0.04×10^3^ M^−1^ [37]. To examine the binding mode of Me-Man for Artocarpin, Banlec, Jacalin, and IPO, the binding position of Me-Man with IPO showed a distant binding site as compared with that for Artocarpin, Banlec, and Jacalin (Figure 7D).

### The N-terminus of IPO is Involved in Tetramerization and Regulates the Carbohydrate Binding Specificity

From ITC results, the binding constant K_A_ of ΔN10IPO to Me-Man and Me-Glc was 3.79×10^4^ M^−1^ and 1.36×10^4^ M^−1^. No interaction between ΔN10IPO and Me-Gal was observed. ΔN10IPO could be recovered as the mannose/glucose specific lectin if ΔN10IPO represented the monomeric IPO and wild-type IPO represented the tetrameric IPO. The monomeric IPO showed 5 times and 6 times binding affinity to Me-Man and Me-Glc, respectively, as compared with those of tetrameric IPO. Therefore, the N terminus of IPO is involved in the carbohydrate recognition, which results in the carbohydrate binding polyspecificity of tetrameric IPO. From the tetrameric IPO structure, the residue Leu5 and His8 in the N terminus of monomer B (chain B) forms 3 hydrogen bonds with the residue Asn19 in the loop between β3 and β4 of monomer A (chain A) (Figure 8). The hydrogen bonds might pull out the loop of β3-β4 and form a larger binding cavity for different carbohydrates in monomer A. However, in ΔN10IPO, the hydrogen bonds would disappear and might relocate the β3-β4 loop to cause a smaller binding cavity. The axial O4 of Me-Gal would not easily enter into the smaller binding cavity. The results might be confirmed by the crystal structure of ΔN10IPO–Me-Man in further study.

In conclusion, we resolved the structures of apo IPO and IPO in complex with Me-Man, Me-Glc and Me-Gal. IPO is proposed to have a tetrameric association by 4 protomers of the β-prism with an additional N terminus, which shows a compact tetrameric association in the JRL family. From gel filtration experiments, we confirmed the tetrameric association of IPO in solution. The N terminus of IPO plays an important role in forming a tetramer. In addition, the binding pocket of IPO was identified and found to bind to Me-Glc, Me-Man, and Me-Gal with similar hydrogen bond networks. Furthermore, the binding constants of IPO were determined by ITC. The IPO structures further extend the diverse quaternary structures of the JRL family of plants and show versatile carbohydrate binding properties regulated by the N terminus. Thus, the wound-inducible protein IPO from sweet potato has versatile carbohydrate binding properties and might play a role in plant defense.

## Materials and Methods

### Materials

The expression vector pTZ18UH containing the IPO gene [GenBank: D89823.1] (pTZ18UH-IPO) of sweet potato (*I. batatas* cv. Tainung 57) was constructed previously [10]. A truncated form of IPO by removing 10 residues of N terminus (pTZ18UH- ΔN10IPO) was amplified by PCR with the primers 5⋅-GCAGGATCCGCCAGATCTGGACCA-3⋅and 5⋅-GTTTTCCCAGTCACGAC-3⋅and further constructed into pTZ18UH. Me-Man (M-9376), Me-Glc (M-6882), Me-Gal (M-1379) and D-galactose (Gal; G0750) were from Sigma-Aldrich (St. Louis, MO). D-mannose (Man; J443) was from Amresco (USA). D-glucose (Glc; GB0219) was from Bio Basic, Canada.

### Protein Expression and Purification

The pTZ18UH-IPO and pTZ18UH-ΔN10IPO vectors were transformed into *Escherichia coli* BL21 (DE3) cells (Novagen). A single colony was cultured in 5 ml LB medium containing 100 µg/ml ampicillin (LB/Amp) at 37°C overnight. The medium was further transferred into 600 ml LB/Amp to an A_600_ of about 0.5 to 0.7 and then induced with 0.1 mM isopropyl-β-D-thiogalactopyranoside (IPTG) at 25°C for 6 hr. Cells harvested by centrifugation were resuspended in a loading buffer (20 mM sodium phosphate, pH 7.4, 0.5 M sodium chloride, 20 mM imidazole). After breaking cells by use of an ultrasonicator (Sonicator 3000, Misonix), the supernatant of the crude cell lysate was loaded onto a Histrap FF column (GE Healthcare) with use of an Äkta Prime fast protein liquid chromatography (FPLC) system (GE Healthcare). After washing the Histrap FF column with 3x column volume of loading buffer (1x phosphate buffered saline, 5 mM adenosine triphosphate, 10 mM MgSO_4_), the IPO protein was eluted by use of elution buffer (50 mM sodium phosphate, pH 7.4, 0.5 M sodium chloride, 500 mM imidazole) with an imidazole gradient. The eluted IPO protein was concentrated and dialysed against a storage buffer (20 mM Tris-HCl, pH 7.0, 10% glycerol) by use of centriplus (Amicon concentrator, Millipore). Quantification of purified proteins involved use of a BioRad protein assay kit (Bio-Rad Laboratories Taiwan Ltd) with bovine serum albumin used as a standard. Finally, the purified IPO protein solution of 3 to 4 mg/ml was used in crystallization.

### Crystallization and Data Collection

Crystallization of the apo IPO protein and IPO in complex with carbohydrates involved the hanging-drop vapor diffusion method at room temperature. The protein solution and buffer of the reservoir was mixed in a 1∶1 volume ratio. The above protein solution of 4 mg/ml IPO was crystallized from a drop containing 0.005 M ferric chloride, 0.05 M sodium citrate pH 5.6, 5% jeffamine M-600 against a reservoir of 0.01 M ferric chloride, 0.1 M sodium citrate pH 5.6, 10% jeffamine M-600. Crystals of apo IPO appeared in 2 days.

IPO complexed with Me-Man, Me-Glc, and Me-Gal involved the co-crystallization method. The protein solution of 3 mg/ml IPO mixed with 10 mM Me-Man was cocrystalized from a drop containing 1.0 M sodium chloride, 5% PEG 6,000 against a reservoir of 2.0 M sodium chloride, 10% PEG 6,000. The crystals of IPO–Me-Man appeared within 3 to 4 days. The protein solution containing 3 mg/ml IPO and 10 mM Me-Glc was co-crystalized from a drop containing 0.05 M sodium acetate, pH 4.6, 0.05 M cadmium chloride, 15% polyethylene glycol 400 (PEG 400) against a reservoir of 0.1 M sodium acetate pH 4.6, 0.1 M cadmium chloride, 30% PEG 400. The crystals of IPO–Me-Glc appeared in 6 to 8 days. The protein solution of 3 mg/ml IPO and 250 mM Me-Gal was co-crystalized from a drop containing 0.2 M sodium formate, 20% w/v polyethylene glycol 3,350 against a reservoir of 0.4 M sodium formate, 40% w/v polyethylene glycol 3,350. The crystals of IPO–Me-Gal appeared in 7 days. A mixture of the reservoir solution with 100% glycerol in a 4∶1 volume ratio was used as cryo-protectant for data collection. The diffraction data were collected at 100K and detected by a Quantum 315 or Quantum 210 CCD detector at the BL13B1 or BL13C1 beamlines of NSRRC (Hsinchu, Taiwan). All diffraction data were processed and scaled with use of the HKL2000 program [38]. The diffraction statistics are in Table 3.

### Structure Determination and Refinement

We used a blastp search for the amino acid sequence of IPO [GenBank: BAA14024.1] against the algorithm of the National Center for Biotechnology Information (NCBI) protein databank database for searching structural templates. The amino acid sequence of *Calystegia sepium* agglutinin (Calsepa), a JRL (PDB: 1OUW), showed 53% sequence identity to that of IPO. The monomeric structure of Calsepa was further used in a search to determine the structure of apo IPO by molecular replacement with use of the program CNS [39]. After cross-rotation and translation of molecular replacement, 4 values were obtained. Initial rigid body refinement for the 4 monomeric structures gave a 48.8% R-factor. Clear continuous electron density could be observed after calculation of Fourier maps, and the 5^th^ molecule of apo IPO was further built accordingly. Because of different space groups for the structures of the IPO–Me-Man, IPO–Me-Glc and IPO–Me-Gal complexes, the resolved monomeric apo IPO was used as a search template in the following molecular replacement method. The solutions of cross-rotation and translation could be obtained with 2 molecules for the IPO–Me-Man complex, 4 molecules for IPO–Me-Glc and 4 molecules for IPO–Me-Gal. Those solutions were further applied to initial rigid body refinement, and reasonable values were obtained (e.g., 36.7% R-factor for IPO–Me-Man, 35.4% for IPO–Me-Glc, and 32.9% for IPO–Me-Gal).

Manual model rebuilding involved use of Coot [40], alternating refinement by the CNS program, with 5% or 10% of the observed reflections randomly selected and set aside for calculation of the R_free_ value. The final refined statistics are in Table 3. For the protein interface of the tetrameric form, IPO–Me-Glc was used as a representative for analysis by the web service PDBe PISA [34]. All molecular representations were prepared with use of DeepView [41] and PyMOL [42]. The coordinates of monomers of apo IPO (e.g., chain A) were subjected to the web service Matras for structure comparison [31].

### Determination of Quaternary Association by Gel Filtration Chromatography

A gel filtration column (Hiload 16/60 Superdex 200 prep grade, GE Healthcare) on an Äkta Prime FPLC system (GE Healthcare) was first equilibrated by a 2x column volume of the running buffer (27 mM Tris-HCl pH 7.0, 2 M NaCl) with or without 0.2 M Me-Glc or 1 M glucose. After equilibration, a 2-ml protein sample containing 0.5 mg/ml IPO in the buffer with or without 0.2 M Me-Glc or 1 M glucose was loaded onto the gel filtration column at a flow rate of 0.6 ml/min. The standard protein markers (Bio-Rad Laboratories Taiwan Ltd) containing 5 mg thyroglobulin (670 kDa), 5 mg gamma-globulin (158 kDa), 5 mg ovalbumin (44 kDa), 2.5 mg myoglobin (17 kDa) and 0.5 mg vitamin B12 (1.35 kDa) were dissolved in 2 ml buffer with or without 0.2 M Me-Glc or 1 M glucose and loaded onto the gel filtration column at a flow rate of 0.6 ml/min. The molecular mass of quaternary association of IPO could be determined by the linear regression equation of the standard protein markers.

### Quantification of Protein and Carbohydrate Solution for Binding Assay

The quantification of protein solution for binding assay was determined by the UV absorption method. The purified IPO protein was dialyzed against 20 mM Tris-HCl, 150 mM NaCl (pH 7.0) at 4°C overnight. The concentration of IPO and ΔN10IPO was determined by UV absorption spectroscopy at 280 nm with the specific extinction coefficient ε of 22,920 M^−1^cm^−1^, which was determined from the prediction of IPO primary sequence. From Beer-Lambert law,

A = ε×b×C

where A is the absorbance of the sample at 280 nm, b is the pathlength in 1 cm, and C is the protein concentration (M).

The protein concentration C could be calculated from the equation. Carbohydrates were prepared by weighting the amount on a microbalance before dissolving in dialysis buffer (20 mM Tris-HCl, 150 mM NaCl pH 7.0).

### Binding Affinity by Isothermal Titration Calorimetry (ITC)

ITC measurements involved use of a MicroCal iTC200 microcalorimeter (GE Healthcare) at 25°C. In individual titration, 1–2 µl carbohydrate solution was added at 180-s intervals by use of a computer-controlled 40 µl syringe to a cell containing 280 µl IPO protein solution under constant stirring at 1,000 rpm. The concentration of IPO protein was 1–3 mM and that of Me-Man, Me-Glc, Me-Gal, Man, Glc and Gal 25–75 mM. The titration of carbohydrate solution in this range of concentration to the dialysis buffer was used as a control. Measurements of the heat change determined from the binding constant (K_A_), reaction stoichiometry (n), and enthalpy (ΔH). The 18 experimental data were fitted for a 1∶1 binding model (one-site of fitting) with Microcal Origin 7.0 software. Free energy (ΔG) and binding entropy (ΔS) were calculated by the equations ΔG = -RTlnK_A_ and ΔG = ΔH – TΔS. R is the gas constant and T the absolute temperature. The optimal c-value in ITC calculation varied between 1 and 10. However, for titrations with Man, Glc and Gal, the c-values were <1.

### Protein Data Bank Accession Codes

The atomic coordinates and structure factors of apo IPO and IPO–carbohydrate structures have been deposited in the RCSB Protein Data bank, with 3R50 for apo IPO, 3R51 for IPO–Me-Man complex, 3R52 for IPO–Me-Glc complex and 4DDN for IPO–Me-Gal complex.

## Supporting Information

Figure S1 **Quaternary structure diversity with the same subunit of β-prism folds in the JRL family.** The quaternary structures in the JRL family can be represented as dimer, tetramer, hexamer, and octomer with the same building block of the β-prism fold. (**A**) Tetramer of Jacalin (PDB: 1UGW), (**B**) dimer of banlec (PDB: 2BMZ), (**C**) tetramer of IPO (this study), (**D**) dimer of calsepa (PDB: 1OUW), (**E**) dimer with 3 repetitive β-prism folds forming hexahedral PPL (PDB: 1ZGS), and (**F**) octomer of heltuba (PDB: 1C3K). The tetramer of Jacalin (**A**) can be easily distinguished from that of IPO (**C**).
(TIF)
