# Correction: Ipomoelin, a Jacalin-Related Lectin with a Compact Tetrameric Association and Versatile Carbohydrate Binding Properties Regulated by Its N Terminus

**DOI:** 10.1371/annotation/5b9b681e-0744-4064-8b57-ed4390b3ce6f

**Published:** 2012-10-04

**Authors:** Wei-Chieh Chang, Kai-Lun Liu, Fang-Ciao Hsu, Shih-Tong Jeng, Yi-Sheng Cheng

The published Funding statement was incorrect. The correct Funding is: Financial supports were from the National Science Council and National Taiwan University, Taiwan, to Y. S. Cheng (NSC 98-2313-B-002-059-MY2 and NTU 10R70614-2) and S. T. Jeng (NSC 99-2313-B-002-005-MY3). The funders had no role in study design, data collection and analysis, decision to publish, or preparation of the manuscript.

