# Supplementary figures and images for: Ipomoelin, a Jacalin-Related Lectin with a Compact Tetrameric Association and Versatile Carbohydrate Binding Properties Regulated by Its N Terminus
Source: PLoS One. 2012 Jul 11;7(7):e40618. doi: 10.1371/journal.pone.0040618 (PMC3394770; doi:10.1371/journal.pone.0040618)

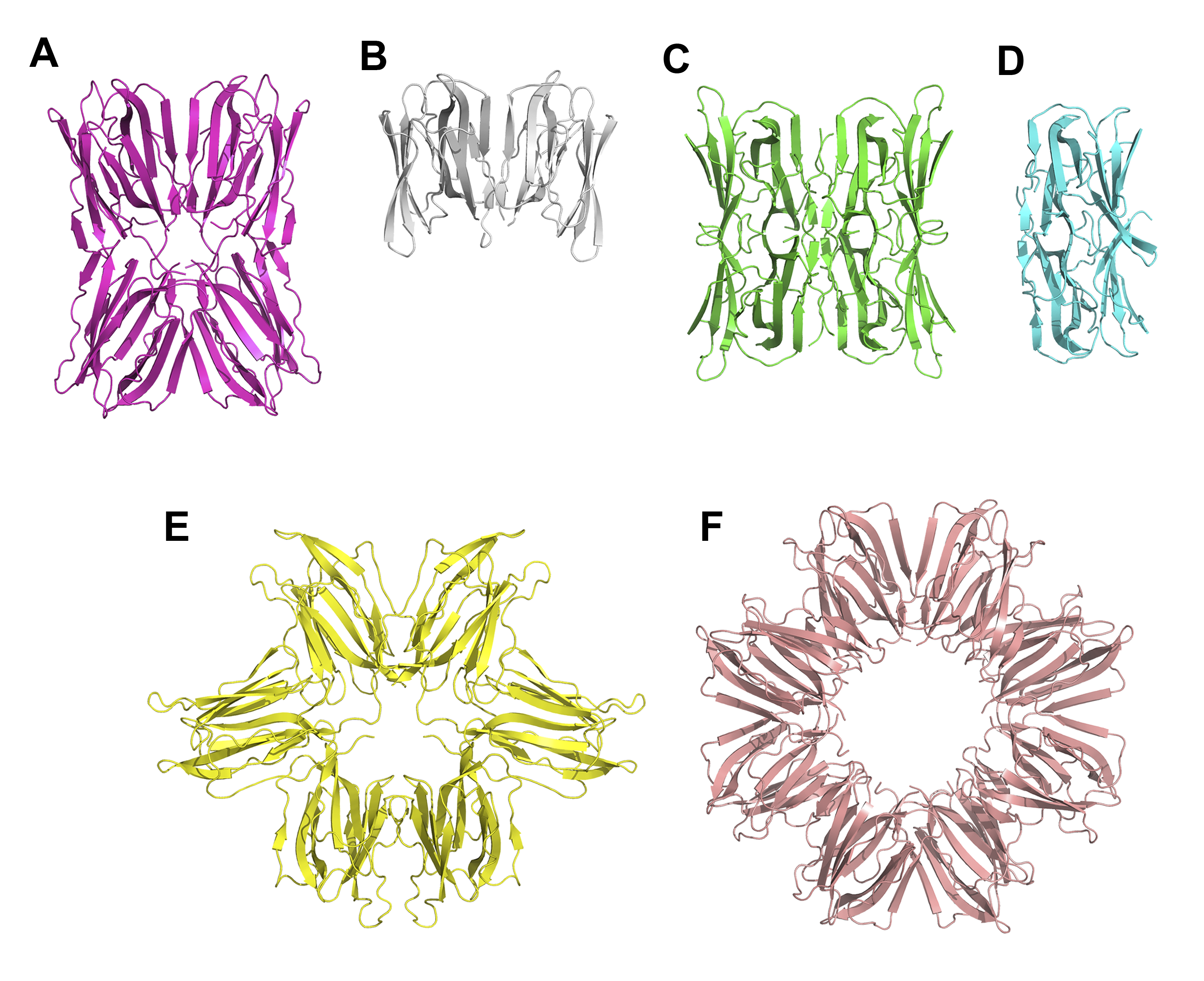

Supplement: Figure S1 — Quaternary structure diversity with the same subunit of β-prism folds in the JRL family. The quaternary structures in the JRL family can be represented as dimer, tetramer, hexamer, and octomer with the same building block of the β-prism fold. (A) Tetramer of Jacalin (PDB: 1UGW), (B) dimer of banlec (PDB: 2BMZ), (C) tetramer of IPO (this study), (D) dimer of calsepa (PDB: 1OUW), (E) dimer with 3 repetitive β-prism folds forming hexahedral PPL (PDB: 1ZGS), and (F) octomer of heltuba (PDB: 1C3K). The tetramer of Jacalin (A) can be easily distinguished from that of IPO (C). (TIF) [file pone.0040618.s001.tif]
